# Identification and Analyzation of Differentially Expressed Transcription Factors in Endometriosis

**DOI:** 10.3389/fmolb.2020.614427

**Published:** 2021-01-07

**Authors:** Shanshan Cong, Qiuyan Guo, Yan Cheng, Jianhua Gao, Liyuan Sun, Jing Wang, Han Wu, Tian Liang, Guangmei Zhang

**Affiliations:** Department of Gynecology, The First Affiliated Hospital, Harbin Medical University, Harbin, China

**Keywords:** endometriosis, transcription factors, infertility, RUNX2, BATF

## Abstract

**Background:** Endometriosis is interpreted as the existence of endometrium outside the uterine cavity, such as ovaries, fallopian tubes and pelvic cavity. Dysmenorrhea, abnormal menstruation, infertility, and chronic pelvic pain are the primary symptoms of endometriosis. Although there are many theories about the origin of endometriosis, the exact factor of the disease has not been confirmed. Therefore, many other mechanisms are still worth exploring.

**Materials and Methods:** The gene lists of the transcription factors (TFs) were selected from the intersections of three databases. The limma R package was used to analyze the differentially expressed genes (DEGs) of GSE6364 and GSE7305 and the DEGs intersected with the TFs to obtain the differentially expressed TFs (DETFs). Subsequently, one-way ANOVA and Student's *t*-test were used to analyze the expression of DETFs in different phases of the endometrium and the endometrium of the infertile and fertile females with endometriosis, respectively. Enrichment analysis and PPI network were performed to reveal the molecular mechanisms of endometriosis. Finally, the plotROC R package was used to evaluate the sensitivity and specificity of hub TFs for the diagnosis of endometriosis.

**Results:** A total of 54 DETFs were screened out in endometriosis. The expression of up-regulated DETFs was gradually increased from the early secretory to the proliferative phase of the endometrium. Most up-regulated DETFs increased expression in the endometrium of infertile females. The pathways of DETFs were mainly enriched in stem cell differentiation, transcription activity, steroid hormone receptor activity and herpes simplex virus. Two hub TFs (RUNX2 and BATF) and two sub-networks were finally acquired from the PPI network. RUNX2 and BATF also had high diagnostic value in endometriosis.

**Conclusion:** We discovered and analyzed 54 DETFs that were closely related to endometriosis, which would contribute to explore new mechanisms of endometriosis and search for new diagnostic markers and effective therapeutic targets.

## Introduction

Endometriosis is a common and highly recurrent gynecologic disease which is characterized by the presence of endometrium tissues outside of the uterus (Bulun, 2009). The aberrant growth of endometriosis can be found in the peritoneal cavity, ovaries, cervix and fallopian tubes leading to pelvic pain, dysmenorrhea and infertility (Garry, 2004). Surgeries of endometriosis constitute the second largest number of surgeries in premenopausal women. The progression of endometriosis is often associated with proliferation, spreading and invasion of endometrial cells in the peritoneal cavity, adaptation of local inflammatory reaction and angiogenesis (Giudice and Kao, 2004). Endometriosis was first described by Von Rokitansky in 1860 (Honda and Katabuchi, 2014). Then, Sampson proposed the most prevalent theory, namely, retrograde menstruation in 1921, which has been highly questioned and challenged (Sampson, 1927). Because endometrial fragment reflux into the peritoneal cavity occurs in 90% of women and only 10% of women have endometriosis (Halme et al., 1984), other causative factors likely play roles in the development and progression of the disease. Although the pathogenesis of endometriosis is largely elusive, many cell migration and invasion-related molecules have been reported to be associated with endometriosis disease progression (Lagana et al., 2019).

Transcription factors (TFs) are a class of proteins that can regulate transcription by binding to the activator or promoter regions of DNA and control gene expression through various mechanisms (Gill, 2001). Previous studies have shown that TFs can play different regulatory roles and are characteristic of high selectivity in different tissues and diseases (Neph et al., 2012). Many researches have exhibited that TFs play a significant role in the occurrence and development of cancer (Frisch et al., 2017; Kotarba et al., 2018; Huh et al., 2019). The transcription products, coding RNAs and non-coding RNAs (ncRNAs), were verified that they could also take part in the process of transcription. Some ncRNAs were closely related to TFs and they could also regulate the function of TFs in some diseases (Feng et al., 2006; Long et al., 2017; Guo et al., 2019). Endometriosis is a sort of benign disease but with the invasion mechanism of malignant tumor. The research of ncRNAs in endometriosis has been extensive, but the research on TFs is still rare. Therefore, it is crucial to study the role of TFs and related pathways in endometriosis which is beneficial for exploring the mechanism of endometriosis and detecting more effective therapeutic targets.

## Materials and Methods

### Data Downloading and Processing

We searched three TFs online datasets and downloaded 1639 TFs from Catalog of Inferred Sequence Binding Preferences (CIS-BP) Database (Weirauch et al., 2014), 1665 TFs from Human Transcription Factor Database (Human TFDB) (Hu et al., 2019), and 1639 TFs from The Human Transcription Factors Database (Lambert et al., 2018). The intersection of TFs from these three databases would be put into our research of endometriosis. We searched “endometriosis” in Gene Expression Omnibus (GEO) (https://www.ncbi.nlm.nih.gov/geo) and acquired four GEO datasets (GSE6364, GSE7305, GSE51981, and GSE120103) after filtrating. The gene symbols of the data were matched with the corresponding GEO platforms (GPL). The raw data of GSE6364, GSE7305 and GSE51981 which applied the GPL570 were reading and normalized by ReadAffy and rma method, respectively, in Affy R package (version 1.62.0) (Gautier et al., 2004) and the data of GSE120103 was chosen series matrix file to download. The version of R used in our research was 3.6.0.

### Identification of DETFs in Endometriosis

We selected the gene expression data of GSE6364 and GSE7305 and divided the data into the endometriosis group and the control group, respectively. Limma R package (version 3.42.2) (Ritchie et al., 2015) was used to perform the differential expression analysis of genes between the endometriosis group and the control group at first. The results were then intersected with TFs from three databases to obtain the co-upregulated DETFs and co-downregulated DETFs.

### Enrichment Analysis

The conversion from gene symbol to Entrez ID before enrichment analysis was applied by org.Hs.eg.db R package (version 3.8.2) (Carlson et al., 2013). The enrichment analysis of Gene Ontology (Go) terms mainly includes three domains: biological process, cellular component and molecular function. ClusterProfiler R package (version 3.12.0) (Yu et al., 2012) was used to carry out the enrichment analysis of Go terms and Kyoto encyclopedia of genes and genomes (KEGG) pathways on DETFs.

### The Establishment and Analysis of Protein-Protein Interaction Network

Protein-protein interaction (PPI) analysis of DETFs was performed base on the STRING online database (https://string-db.org) which integrates the interaction information of multitudinous proteins. Cytoscape (version 3.7.1) was used to customize and analyze the PPI network. The cytoHubba app (Chin et al., 2014) in Cytoscape was used for calculating the hub TFs by MCC algorithm and the MCODE app (Bader and Hogue, 2003) was applied to set up the sub-network.

### Statistical Analysis

The comparison method between the expression of DETFs in infertile and fertile endometriosis was Student's *t*-test. The one-way analysis of variance (one-way ANOVA) was used to compare the expression of DETFs in early secretory, mid-secretory and proliferative phase of endometrium in endometriosis. The plotROC R package (version 2.2.1) (Sachs, 2017) was used to evaluate the sensitivity and specificity of TF in the diagnosis of endometriosis. All cut-off *p*-value was set as *P* < 0.05 in our research.

## Results

### Identification of DETFs in Endometriosis

**Figure 1:**
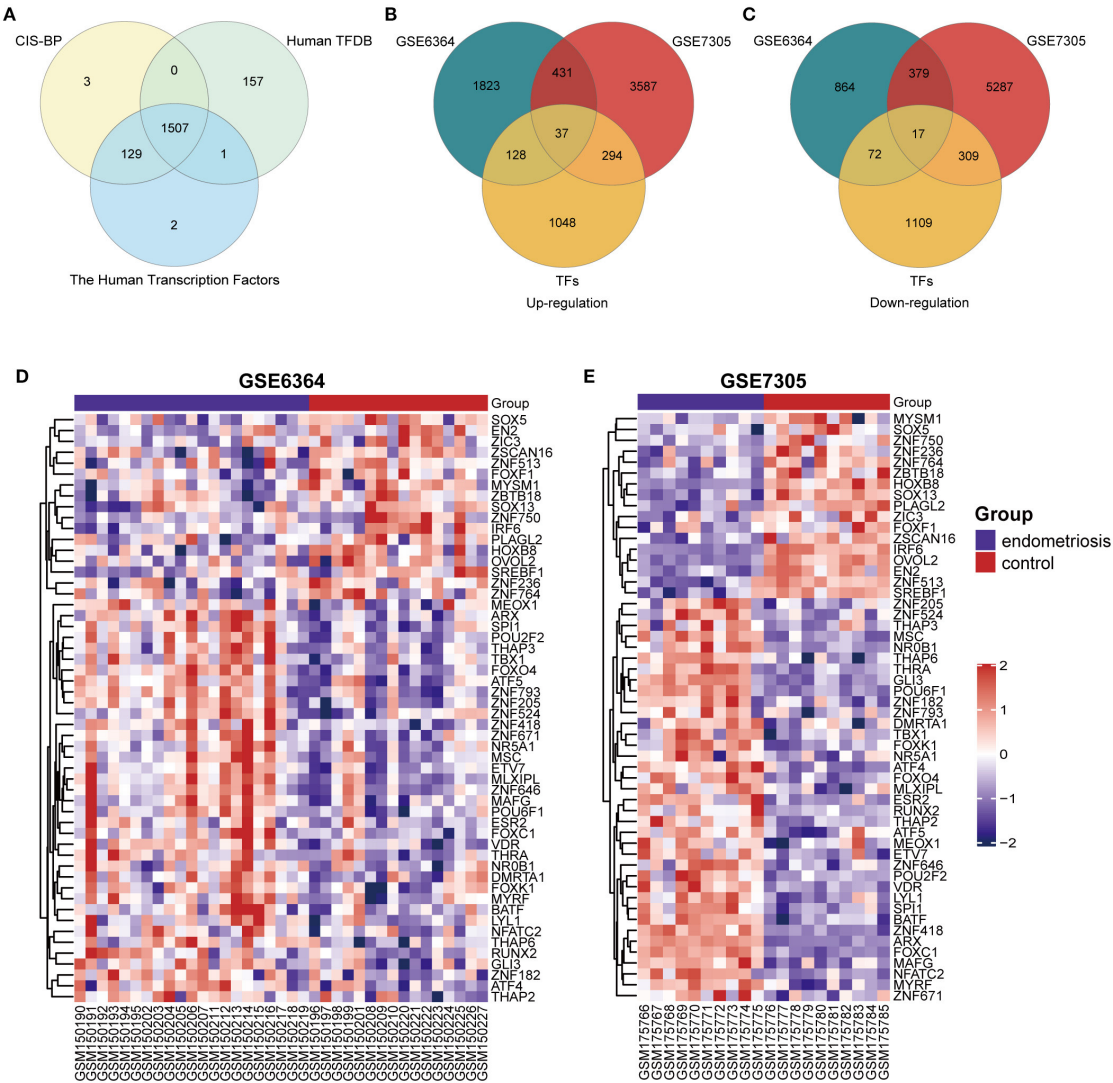
Identification of 54 DETFs in endometriosis. **(A)** Identification of 1507 TFs from three TFs databases (CIS-BP, Human TFDB and The Human Transcription Factors). **(B)** Identification of 37 up-regulated DETFs from GSE6364 and GSE7305. **(C)** Identification of 17 down-regulated DETFs from GSE6364 and GSE7305. **(D)** The heatmap of 54 DETFs in GSE6364. Each row represents a TF and each column represents a sample. **(E)** The heatmap of 54 DETFs in GSE7305. Each row represents a TF and each column represents a sample.

A total of 1,507 TFs were collected from the intersection of three TFs databases (CIS-BP, Human TFDB and The Human Transcription Factors) (Figure 1A). Then the gene expression datasets of endometrial samples with or without endometriosis (GSE6364 and GSE7305) were normalized and removed batch effect. We acquired 3,751 DEGs from GSE6364 and 10,341 DEGs from GSE7305 by limma R package. The results were then intersected with 1,507 TFs and 54 DETFs (37 up-regulated DETFs and 17 down-regulated DETFs) were obtained in endometriosis at the end (Figures 1B,C). The list of 54 DETFs was exhibited in Table 1. From the heatmap of 54 DETFs in GSE6364 and GSE7305, we found the samples could be divided into endometriosis and control group distinctly (Figures 1D,E).

**Table 1:** The exhibition of 54 DETFs (37 up-regulated DETFs and 17 down-regulated DETFs) in endometriosis.

**DETFs**	**TF lists**
Up-regulated	FOXO4, MLXIPL, SPI1, ZNF205, VDR, POU2F2, ZNF793, ETV7, ZNF418, ZNF671, ARX, MSC, FOXC1, ZNF646, TBX1, ATF5, THAP6, POU6F1, MAFG, FOXK1, MYRF, THAP3, ZNF182, THAP2, ATF4, MEOX1, LYL1, NR5A1, RUNX2, GLI3, DMRTA1, ESR2, ZNF524, NR0B1, NFATC2, BATF, and THRA
Down-regulated	SOX5, IRF6, EN2, HOXB8, ZBTB18, SREBF1, ZNF764, OVOL2, SOX13, FOXF1, PLAGL2, ZNF750, MYSM1, ZSCAN16, ZIC3, ZNF513, and ZNF236

### The Expression of DETFs in Different Phases of the Endometrium

**Figure 2:**
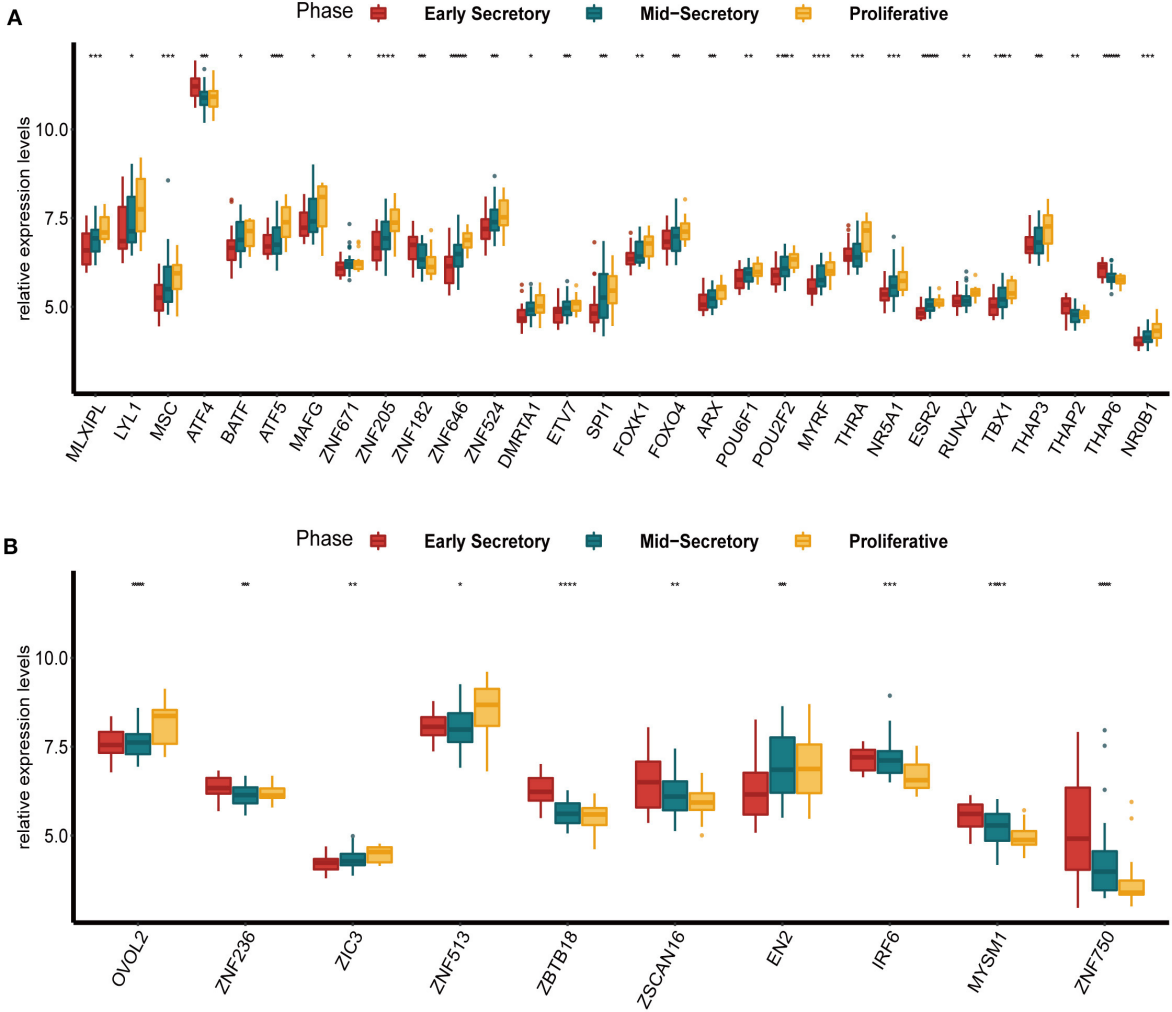
The expression of DETFs in different phases (early secretory phase, mid-secretory phase and proliferative phase) of the endometrium with endometriosis. **(A)** The expression of up-regulated DETFs in different phases of the endometrium. **(B)** The expression of down-regulated DETFs in different phases of the endometrium. (The statistical method between multivariate groups was the one-way ANOVA. *, *P* < 0.05; **, *P* < 0.01; ***, *P* < 0.001; ****, *P* < 0.0001).

To examine the expression of DETFs in different phases of endometrium in endometriosis, we found a GEO dataset (GSE51981) which contained the expression of TFs in the early secretory phase (15–19 days of the menstrual cycle), the mid-secretory phase (20–23 days of the menstrual cycle) and the proliferative phase (5–14 days of the menstrual cycle) of endometrium in endometriosis. We compared the expression of up-regulated DETFs and down-regulated DETFs in these three phases of endometrium, respectively. We found the expression of almost all up-regulated DETFs were gradually increased from the early secretory and the mid-secretory to the proliferative phase with *P* < 0.05 (Figure 2A). On the contrary, the expression of down-regulated DETFs was gradually decreased from the early secretory and the mid-secretory to the proliferative phase with *P* < 0.05 (Figure 2B).

### The Expression of DETFs in the Endometrium of the Infertile and Fertile Females

**Figure 3:**
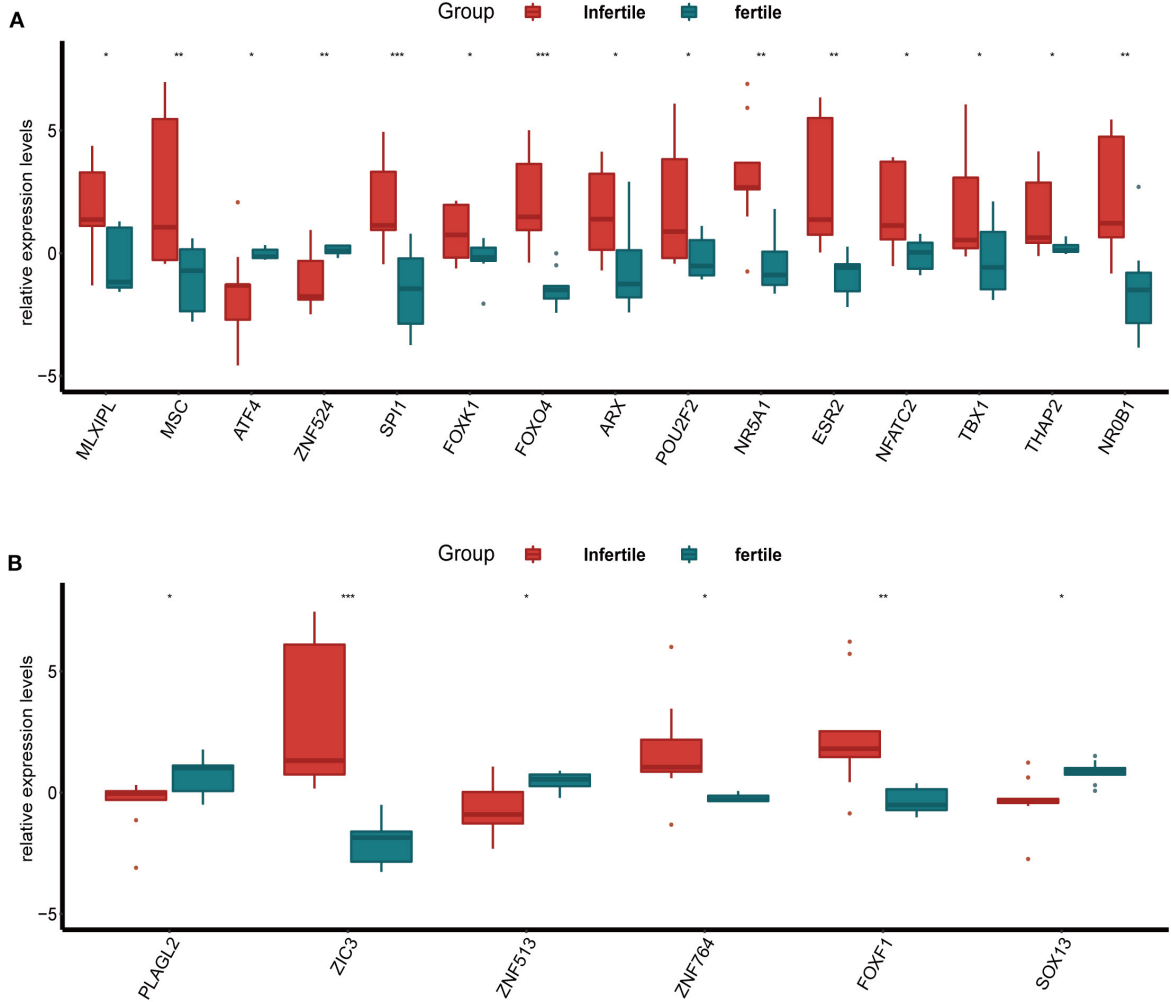
The expression of DETFs in the endometrium of the infertile and fertile females with endometriosis. **(A)** The expression of up-regulated DETFs in the endometrium of the infertile and fertile females. **(B)** The expression of down-regulated DETFs in the endometrium of the infertile and fertile females. (The statistical method between the two groups was Student's *t*-test. *, *P* < 0.05; **, *P* < 0.01; ***, *P* < 0.001; ****, *P* < 0.0001).

Furthermore, we compared the expression of up-regulated DETFs and down-regulated DETFs in the endometrium of the infertile and fertile females with endometriosis in GSE120103. The results showed that the majority of up-regulated DETFs increased expression in the endometrium of the infertile females with *P* < 0.05 (Figure 3A). However, the down-regulated DETFs exhibited decreased expression in the endometrium of the infertile females with *P* < 0.05 (Figure 3B). Strangely, the ATF4 exhibited a reverse trend with up-regulated DETFs in different condition of the endometrium.

### The Enrichment Analysis of DETFs

**Figure 4:**
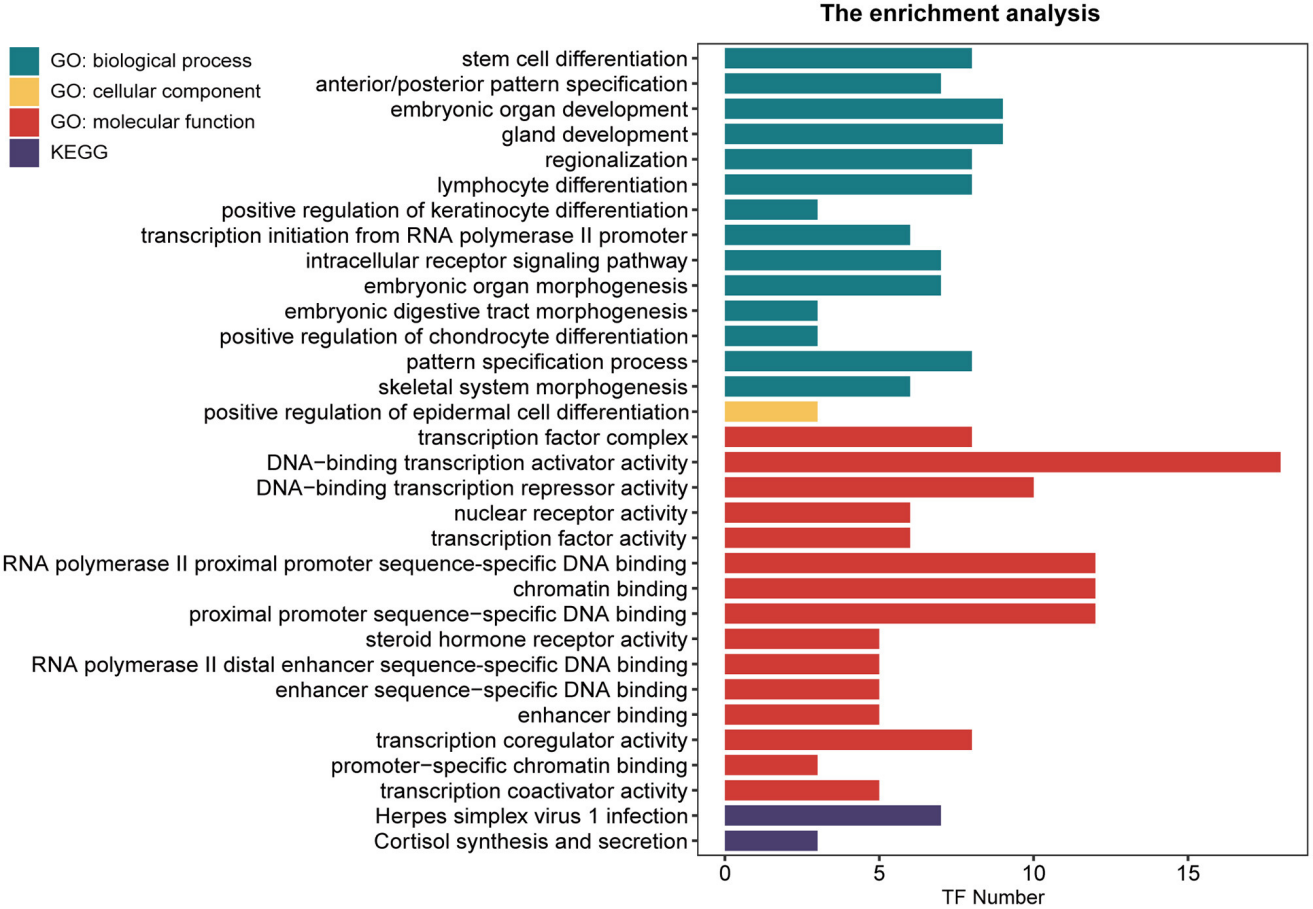
The Go terms and KEGG pathways enrichment analysis of 54 DETFs in endometriosis. The Go terms conclude the biological process, cellular component and molecular function. The horizontal axis represents the number of TFs enriched in each term (*P* < 0.05).

To further study the 54 DETFs, Go terms enrichment analysis and KEGG pathways enrichment analysis were implemented. The Go function of the DETFs was mainly enriched on stem cell differentiation, transcription factor complex, DNA-binding transcription activator activity, DNA-binding transcription repressor activity, and steroid hormone receptor activity. The KEGG pathways of DETFs were enriched on herpes simplex virus, and cortisol synthesis and secretion (Figure 4).

### Identification of Hub TFs and Sub-networks

**Figure 5:**
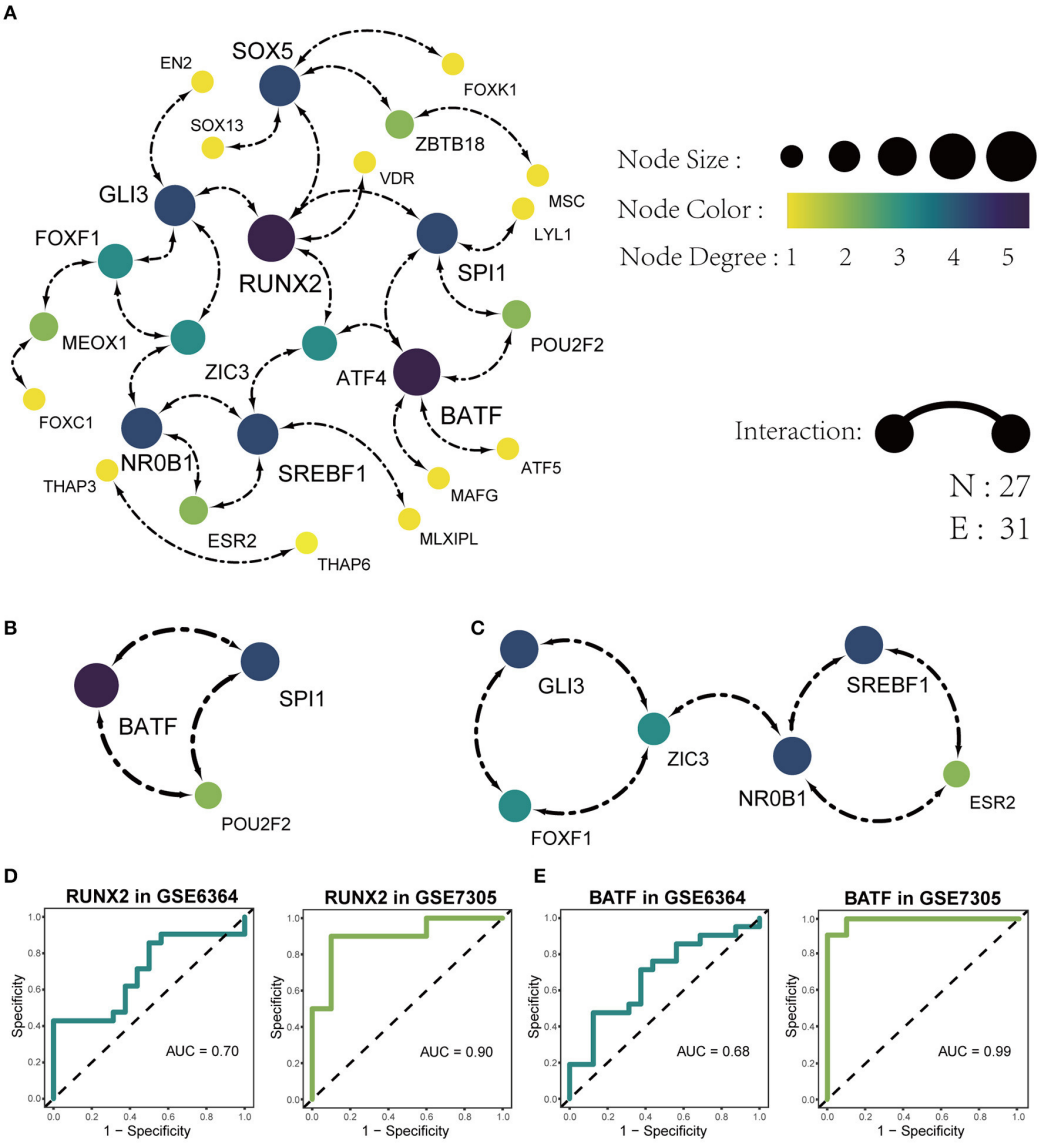
Identification of PPI networks with 54 DETFs. **(A)** The construction of the PPI network concluding 27 nodes and 31 edges. Each node represents a protein and each edge represents the interaction between two proteins. **(B)** Identification of BATF-SPI1-POU2F2 sub-network by MODE app in Cytoscape. **(C)** Identification of GLI3-FOXF1-ZIC3-NR0B1-ESR2-SREBF1 sub-network by MODE app in Cytoscape. **(D)** The ROC analysis of the sensitivity and specificity for the diagnosis of endometriosis based on RUNX2 in GSE6364 (left) and GSE7305 (right). **(E)** The ROC analysis of the sensitivity and specificity for the diagnosis of endometriosis based on BATF in GSE6364 (left) and GSE7305 (right).

The list of 54 DETFs was uploaded to STRING online database and then the PPI network was visualized on these DETFs by Cytoscape. The finally PPI network concluded 27 nodes and 31 edges (Figure 5A). Two hub TFs (RUNX2 and BATF) were picked out from 27 nodes by the MCC algorithm in cytoHubba of Cytoscape (The filtered score of hub TF was ≥5). We also acquired two sub-networks (BATF-SPI1-POU2F2 and GLI3-FOXF1-ZIC3-NR0B1-ESR2-SREBF1) from the PPI network of 54 DETFs (Figures 5B,C). The Receiver Operating Characteristic (ROC) analysis was used to evaluate the sensitivity and specificity of RUNX2 and BATF for the diagnosis of endometriosis. The Area Under Curve (AUC) of RUNX2 in GSE6364 and GSE7305 was 0.70 and 0.90, respectively (Figure 5D). The AUC of BATF in GSE6364 and GSE7305 was 0.68 and 0.99 respectively (Figure 5E). These results meant that RUNX2 and BATF had certain value in diagnosing endometriosis.

## Discussion

Endometriosis is characterized by chronic pain, infertility and recurrence, which causing long-term physical and psychological effects on women. Although endometriosis is a kind of benign disease, the growth pattern of it is very similar to malignant tumor. However, the exact cause of endometriosis is still unclear and the current treatments of endometriosis always have some flaws. Previous studies on endometriosis have mainly focused on genes, microRNAs, lncRNAs or circRNAs. Nevertheless, the research of TFs which could participate in regulating the process of transcription from genes to above-mentioned RNAs was limited. As a group of proteins, TFs' function is not limited to transcription regulation, but also can be used as drug targets which could play a crucial role in the treatment of cancer or other diseases (Gronemeyer et al., 2004; Overington et al., 2006). Therefore, it is extremely urgent to study the expression of TFs in endometriosis and it is beneficial to seek drug targets for the therapy of endometriosis.

With the rapid development of the high-throughput detection techniques and various databases, more and more researches are focus on bioinformatics analysis which can also be the basics of molecular biology experiment. This research mainly employed bioinformatics methods to identify DETFs in endometriosis and analyze the expression of DETFs both in different phases of the endometrium and in the endometrium of the infertile and fertile females. The enrichment analysis and PPI also were performed on DETFs to explore valuable hub TFs and pathways in endometriosis. To get accurate gene names of TFs, we searched three TFs databases (CIS-BP, Human TFDB and The Human Transcription Factors). In total, 1,507 repetitive TFs were selected from these three databases. We also chose two GEO datasets to perform the differential analysis of genes and got the intersection of the above two DEGs and 1,507 TFs. Eventually, we got 54 DETFs concluding 37 up-regulated DETFs and 17 down-regulated DETFs to implement the further analysis.

In different phases of the endometrium with endometriosis, the up-regulated DETFs exhibited the highest expression in the proliferative phase and the lowest expression in the early secretory phase of the endometrium. However, the down-regulated DETFs presented the opposite situation. We considered that this phenomenon might have something to do with the levels of various hormones in different phases of the endometrium. In the proliferative phase, the estrogen plays a leading role and in the mid-secretory phase, the progestin has a peak and estrogen has a second peak. In endometriosis, the balance between estrogen and progesterone is interrupted, exhibiting the phenomenon of estrogen dominance and progesterone resistance (Marquardt et al., 2019). Estrogen is characterized by promoting epithelial proliferation and some DETFs are exactly associated with the cell differentiation and DNA transcription in the result of enrichment analysis which could affect epithelial proliferation (Boxer et al., 2014). On the contrary, progestin could restrain the proliferation and block estrogen-induced DNA synthesis (Pan et al., 2006), but the decrease of DETFs expression was not as obvious as the early secretory phase which might be due to the second peak of estrogen. Meanwhile, these hormones all show low levels in the early secretory phase which may cause that the up-regulated DETFs exhibit the lowest expression in this phase.

In infertile or fertile females with endometriosis, almost all up-regulated DETFs were high expression in infertile females and only six down-regulated DETFs were differentially expressed in these two groups. In addition to the effects of hormones on infertility (Practice Committee of the American Society for Reproductive M, 2006), many other factors [such as inflammation, immune, growth and angiogenic factors, and aberrantly expressed genes (Gupta et al., 2008; Macer and Taylor, 2012)] are also involved. These factors might affect the expression of TFs, or it can be said that they and TFs were co-expressed to cause infertility simultaneously. André GM et al. verified that FOXP3 polymorphisms, a sort of TFs, had an association with endometriosis and idiopathic infertility which would demonstrate that endometriosis was a kind of autoimmune disease (Andre et al., 2011). Another TF, STAT4, was also been linked to endometriosis-related infertility by Zamani et al. (2016). In our research, NR5A1 was high expression in the infertile females with endometriosis and it had the ability to transfer an endometrial stromal cell to an endometriotic-like cell with GATA6 simultaneously by the study of Bernardi et al. (2019). In the meantime, ESR2 also exhibited high expression and studies had proved that ESR2 could influence susceptibility to endometriosis with infertility (Lamp et al., 2011).

However, some DETFs (such as ATF4 and ZIC3) showed the opposite tendency from the same group DETFs in the above two analyses. But the results were supported by relevant researches. ATF4 participated in the process that the endoplasmic reticulum stress inducing by dienogest suppressed the proliferation and invasiveness of endometriotic stromal cells (Choi et al., 2020). From the research, we could assume that the high expression of ATF4 might play a role in inhibiting the proliferation of endometriosis. This was consistent with our findings that ATF4 was high expression in the non-proliferative phase and low expression in the proliferative phase. In the research of Brenjian et al. the expression level of ATF4 elevated in the course of resveratrol treating patients with PCOS which was an important cause of infertility (Brenjian et al., 2020). So was our study, ATF4 had a higher level in the fertile than infertile patients with endometriosis.

We summed up the results of Go terms enrichment analysis on 54 DETFs and found that the Go terms were mainly enriched in four aspects (the differentiation of stem cell, lymphocyte and epidermal cell, embryonic development, DNA transcription, and transcription factor related terms). These terms were closely related to the proliferation of the endometrium. Therefore, the research of TFs on endometrial related diseases was essential. Maybe we could start with DETFs to study the targets for treating endometriosis. At the same time, the KEGG pathways linking to 54 DETFs were herpes simplex virus(HSV) 1 infection and cortisol synthesis and secretion. As we have known, HSV is the chief culprit of the infection about oro-facial areas and genital tracts. But Farsimadan et al. considered that HSV correlated with infertility (Farsimadan and Motamedifar, 2020). Therefore, affecting the endometrium might be one reason for HSV causing females infertility. Another KEGG pathway was cortisol synthesis and secretion, which signified that DETFs took part in the process of cortisol synthesis and secretion. Too much cortisol could inhibit pituitary gonadotropin and cause menstrual irregularity (Newell-Price et al., 2006). Thiruchelvam et al. had proved that cortisol played a role in menses and endometrial repair by regulating angiogenesis (Thiruchelvam et al., 2016). These researches explained that there were indeed differences in the expression of TFs in different phases of the endometrium by affecting the synthesis and secretion of sex hormones.

By the PPI analysis, we found two hub TFs (RUNX2 and BATF) and they had significant differences in the analysis of different phases of the endometrium and the analysis of infertile and fertile females with endometriosis. At the same time, two sub-networks were recognized and one of the sub-networks was BATF-SPI1-POU2F2. The BATF-SPI1-POU2F2 happened to contain BATF hub TF. We found the sub-network was associated with lymphocyte differentiation, leukocyte differentiation and DNA-binding transcription activator activity. The differentiation of lymphocytes and white blood cells is mainly stimulated by inflammation, which also confirmed that inflammation was closely related to the pathophysiology of endometriosis (Samimi et al., 2019).

RUNX2, a member of the Runx family, encodes a nuclear protein that plays a key role in osteoblastic differentiation and skeletal morphogenesis. Researches had shown that RUNX2 participates in the processing of stromal proliferation, differentiation, and remodeling in the course of decidualization (Athilakshmi et al., 2009). Gellersen et al. considered that aberrant decidualization was involved in the pathogenesis of endometriosis (Gellersen and Brosens, 2014). The decidualization of endometrial stromal cells in endometriosis was confirmed to be destroyed (Aghajanova et al., 2010). Heitmann et al. also verified that the impairment of normal decidual response correlated with implantation failure and menstrual disturbances in endometriosis (Heitmann et al., 2014). BATF is a member of the AP-1 superfamily of transcription factors. Studies had shown that BATF could play a crucial role in lymphocyte, such as the CD8+ T cell (Kurachi et al., 2014), the CD4+ T cell (Li et al., 2012) and follicular helper T cells (Sahoo et al., 2015) which are all important cells in the immune system and are closely related to inflammation. Therefore, we had reasons to speculate that RUNX2 and BATF also played a role in the mechanism of endometriosis. At the same time, we calculated the diagnostic value of two hub DETFs (RUNX2 and BATF) for endometriosis. The results showed that RUNX2 and BATF were characterized by high specificity and sensitivity in the diagnostic of endometriosis. This could further confirm the role of hub DETFs in endometriosis and help us discover new biomarkers for endometriosis in the future.

## Conclusion

We screened out 54 DETFs and found the majority of DETFs exhibited abnormal expression in different phases of the endometrium and infertile or fertile females with endometriosis. Meanwhile, the GO terms and KEGG pathways related to DETFs of endometriosis mainly enriched in stem cell differentiation, transcription activity, steroid hormone receptor activity and herpes simplex virus. Besides, two hub TFs and two sub-networks were discovered in our research. The high diagnostic value of RUNX2 and BATF had also been exhibited. The results of our research would benefit to discover new mechanisms of endometriosis and lay the foundation for detecting new biomarkers and effective therapeutic targets for endometriosis. In the future, we still need more experiments to verify the results.

## Data Availability Statement

Publicly available datasets were analyzed in this study. This data can be found at: https://www.ncbi.nlm.nih.gov/geo.

## Author Contributions

GZ designed and directed all the research. SC, QG, YC, JG, LS, JW, HW, and TL performed the data processing and experimental analysis. GZ, SC, and QG drafted the manuscript. All authors reviewed and approved the final version of the manuscript.

## Conflict of Interest

The authors declare that the research was conducted in the absence of any commercial or financial relationships that could be construed as a potential conflict of interest.

## References

[B1] Aghajanova L. Horcajadas J. A. Weeks J. L. Esteban F. J. Nezhat C. N. Conti M.. (2010). The protein kinase A pathway-regulated transcriptome of endometrial stromal fibroblasts reveals compromised differentiation and persistent proliferative potential in endometriosis. Endocrinology 151, 1341–1355. 10.1210/en.2009-0923 20068008 PMC2840687

[B2] Andre G. M. Barbosa C. P. Teles J. S. Vilarino F. L. Christofolini D. M. Bianco B. (2011). Analysis of FOXP3 polymorphisms in infertile women with and without endometriosis. Fertil. Steril. 95, 2223–2227. 10.1016/j.fertnstert.2011.03.033 21481380

[B3] Athilakshmi K. Li Q. Bagchi M. K. Bagchi I. C. (2009). The transcription factor Runx2 functions downstream of BMP2 to regulate decidualization in the mouse. Biol. Reprod. 81, 415–415. 10.1093/biolreprod/81.s1.41519357367

[B4] Bader G. D. Hogue C. W. (2003). An automated method for finding molecular complexes in large protein interaction networks. BMC Bioinform. 4:2. 10.1186/1471-2105-4-2 12525261 PMC149346

[B5] Bernardi L. A. Dyson M. T. Tokunaga H. Sison C. Oral M. Robins J. C.. (2019). The essential role of GATA6 in the activation of estrogen synthesis in endometriosis. Reprod. Sci. 26, 60–69. 10.1177/1933719118756751 29402198 PMC6344952

[B6] Boxer L. D. Barajas B. Tao S. Zhang J. Khavari P. A. (2014). ZNF750 interacts with KLF4 and RCOR1, KDM1A, and CTBP1/2 chromatin regulators to repress epidermal progenitor genes and induce differentiation genes. Genes Dev. 28, 2013–2026. 10.1101/gad.246579.114 25228645 PMC4173152

[B7] Brenjian S. Moini A. Yamini N. Kashani L. Faridmojtahedi M. Bahramrezaie M.. (2020). Resveratrol treatment in patients with polycystic ovary syndrome decreased pro-inflammatory and endoplasmic reticulum stress markers. Am. J. Reprod. Immunol. 83:e13186. 10.1111/aji.13186 31483910

[B8] Bulun S. E. (2009). Endometriosis. N. Engl. J. Med. 360, 268–279. 10.1056/NEJMra0804690 19144942

[B9] Carlson M. Falcon S. Pages H. Li N. (2013). org. Hs. eg. db: Genome wide annotation for Human. R Package Version, 3.

[B10] Chin C. H. Chen S. H. Wu H. H. Ho C. W. Ko M. T. Lin C. Y. (2014). cytoHubba: identifying hub objects and sub-networks from complex interactome. BMC Syst. Biol. 8(Suppl. 4):S11. 10.1186/1752-0509-8-S4-S11 25521941 PMC4290687

[B11] Choi J. Jo M. Lee E. Lee D. Y. Choi D. (2020). Dienogest regulates apoptosis, proliferation, and invasiveness of endometriotic cyst stromal cells via endoplasmic reticulum stress induction. Mol. Hum. Reprod. 26, 30–39. 10.1093/molehr/gaz064 31814016

[B12] Farsimadan M. Motamedifar M. (2020). The effects of human immunodeficiency virus, human papillomavirus, herpes simplex virus-1 and −2, human herpesvirus-6 and −8, cytomegalovirus, and hepatitis B and C virus on female fertility and pregnancy. Br. J. Biomed. Sci. 1–11. 10.1080/09674845.2020.1803540 32726192

[B13] Feng J. Bi C. Clark B. S. Mady R. Shah P. Kohtz J. D. (2006). The Evf-2 noncoding RNA is transcribed from the Dlx-5/6 ultraconserved region and functions as a Dlx-2 transcriptional coactivator. Genes Dev. 20, 1470–1484. 10.1101/gad.1416106 16705037 PMC1475760

[B14] Frisch S. M. Farris J. C. Pifer P. M. (2017). Roles of Grainyhead-like transcription factors in cancer. Oncogene 36, 6067–6073. 10.1038/onc.2017.178 28714958

[B15] Garry R. (2004). Is insulin resistance an essential component of PCOS?: the endometriosis syndromes: a clinical classification in the presence of aetiological confusion and therapeutic anarchy. Hum. Reprod. 19, 760–768. 10.1093/humrep/deh147 15033944

[B16] Gautier L. Cope L. Bolstad B. M. Irizarry R. A. (2004). Affy–analysis of affymetrix geneChip data at the probe level. Bioinformatics 20, 307–315. 10.1093/bioinformatics/btg405 14960456

[B17] Gellersen B. Brosens J. J. (2014). Cyclic decidualization of the human endometrium in reproductive health and failure. Endocr. Rev. 35, 851–905. 10.1210/er.2014-1045 25141152

[B18] Gill G. (2001). Regulation of the initiation of eukaryotic transcription. Essays Biochem. 37, 33–43. 10.1042/bse0370033 11758455

[B19] Giudice L. C. Kao L. C. (2004). Endometriosis. Lancet 364, 1789–1799. 10.1016/S0140-6736(04)17403-5 15541453

[B20] Gronemeyer H. Gustafsson J. A. Laudet V. (2004). Principles for modulation of the nuclear receptor superfamily. Nat. Rev. Drug Discov. 3, 950–964. 10.1038/nrd1551 15520817

[B21] Guo Q. He Y. Sun L. Kong C. Cheng Y. Wang P.. (2019). Identification of potential prognostic TF-associated lncRNAs for predicting survival in ovarian cancer. J. Cell. Mol. Med. 23, 1840–1851. 10.1111/jcmm.14084 30549251 PMC6378234

[B22] Gupta S. Goldberg J. M. Aziz N. Goldberg E. Krajcir N. Agarwal A. (2008). Pathogenic mechanisms in endometriosis-associated infertility. Fertil. Steril. 90, 247–257. 10.1016/j.fertnstert.2008.02.093 18672121

[B23] Halme J. Hammond M. G. Hulka J. F. Raj S. G. Talbert L. M. (1984). Retrograde menstruation in healthy women and in patients with endometriosis. Obstetr. Gynecol. 64, 151–154. 6234483

[B24] Heitmann R. J. Langan K. L. Huang R. R. Chow G. E. Burney R. O. (2014). Premenstrual spotting of >/=2 days is strongly associated with histologically confirmed endometriosis in women with infertility. Am. J. Obstet. Gynecol. 211, 358.e351–e356. 10.1016/j.ajog.2014.04.041 24799313

[B25] Honda R. Katabuchi H. (2014). “Pathological aspect and pathogenesis of endometriosis,” in Endometriosis, ed T. Harada (Springer), 9–18.

[B26] Hu H. Miao Y. R. Jia L. H. Yu Q. Y. Zhang Q. Guo A. Y. (2019). AnimalTFDB 3.0: a comprehensive resource for annotation and prediction of animal transcription factors. Nucleic Acids Res. 47, D33–D38. 10.1093/nar/gky822 30204897 PMC6323978

[B27] Huh H. D. Kim D. H. Jeong H. S. Park H. W. (2019). Regulation of TEAD transcription factors in cancer biology. Cells 8:600. 10.3390/cells8060600 31212916 PMC6628201

[B28] Kotarba G. Krzywinska E. Grabowska A. I. Taracha A. Wilanowski T. (2018). TFCP2/TFCP2L1/UBP1 transcription factors in cancer. Cancer Lett. 420, 72–79. 10.1016/j.canlet.2018.01.078 29410248

[B29] Kurachi M. Barnitz R. A. Yosef N. Odorizzi P. M. DiIorio M. A. Lemieux M. E.. (2014). The transcription factor BATF operates as an essential differentiation checkpoint in early effector CD8+ T cells. Nat. Immunol. 15, 373–383. 10.1038/ni.2834 24584090 PMC4000237

[B30] Lagana A. S. Garzon S. Gotte M. Vigano P. Franchi M. Ghezzi F.. (2019). The pathogenesis of endometriosis: molecular and cell biology insights. Int. J. Mol. Sci. 20:5615. 10.3390/ijms20225615 31717614 PMC6888544

[B31] Lambert S. A. Jolma A. Campitelli L. F. Das P. K. Yin Y. Albu M. (2018). The human transcription factors. Cell 172, 650–665. 10.1016/j.cell.2018.01.029 29425488 PMC12908702

[B32] Lamp M. Peters M. Reinmaa E. Haller-Kikkatalo K. Kaart T. Kadastik U.. (2011). Polymorphisms in ESR1, ESR2 and HSD17B1 genes are associated with fertility status in endometriosis. Gynecol. Endocrinol. 27, 425–433. 10.3109/09513590.2010.495434 20586553

[B33] Li P. Spolski R. Liao W. Wang L. Murphy T. L. Murphy K. M.. (2012). BATF-JUN is critical for IRF4-mediated transcription in T cells. Nature 490, 543–546. 10.1038/nature11530 22992523 PMC3537508

[B34] Long Y. Wang X. Youmans D. T. Cech T. R. (2017). How do lncRNAs regulate transcription? Sci. Adv. 3:eaao2110. 10.1126/sciadv.aao2110 28959731 PMC5617379

[B35] Macer M. L. Taylor H. S. (2012). Endometriosis and infertility: a review of the pathogenesis and treatment of endometriosis-associated infertility. Obstet. Gynecol. Clin. North Am. 39, 535–549. 10.1016/j.ogc.2012.10.002 23182559 PMC3538128

[B36] Marquardt R. M. Kim T. H. Shin J. H. Jeong J. W. (2019). Progesterone and estrogen signaling in the endometrium: what goes wrong in endometriosis? Int. J. Mol. Sci. 20:3822. 10.3390/ijms20153822 31387263 PMC6695957

[B37] Neph S. Stergachis A. B. Reynolds A. Sandstrom R. Borenstein E. Stamatoyannopoulos J. A. (2012). Circuitry and dynamics of human transcription factor regulatory networks. Cell 150, 1274–1286. 10.1016/j.cell.2012.04.040 22959076 PMC3679407

[B38] Newell-Price J. Bertagna X. Grossman A. B. Nieman L. K. (2006). Cushing's syndrome. Lancet 367, 1605–1617. 10.1016/S0140-6736(06)68699-6 16698415

[B39] Overington J. P. Al-Lazikani B. Hopkins A. L. (2006). How many drug targets are there? Nat. Rev. Drug Discov. 5, 993–996. 10.1038/nrd2199 17139284

[B40] Pan H. Deng Y. Pollard J. W. (2006). Progesterone blocks estrogen-induced DNA synthesis through the inhibition of replication licensing. Proc. Natl. Acad. Sci. U.S.A. 103, 14021–14026. 10.1073/pnas.0601271103 16966611 PMC1599905

[B41] Practice Committee of the American Society for Reproductive M (2006). Endometriosis and infertility. Fertil. Steril. 86, S156–S160. 10.1016/j.fertnstert.2006.08.014 17055813

[B42] Ritchie M. E. Phipson B. Wu D. Hu Y. Law C. W. Shi W.. (2015). limma powers differential expression analyses for RNA-sequencing and microarray studies. Nucleic Acids Res. 43:e47. 10.1093/nar/gkv007 25605792 PMC4402510

[B43] Sachs M. C. (2017). plotROC: a tool for plotting ROC curves. J. Stat. Softw. 79:2. 10.18637/jss.v079.c02 30686944 PMC6347406

[B44] Sahoo A. Alekseev A. Tanaka K. Obertas L. Lerman B. Haymaker C.. (2015). Batf is important for IL-4 expression in T follicular helper cells. Nat. Commun. 6:7997. 10.1038/ncomms8997 26278622 PMC4557271

[B45] Samimi M. Pourhanifeh M. H. Mehdizadehkashi A. Eftekhar T. Asemi Z. (2019). The role of inflammation, oxidative stress, angiogenesis, and apoptosis in the pathophysiology of endometriosis: basic science and new insights based on gene expression. J. Cell. Physiol. 234, 19384–19392. 10.1002/jcp.28666 31004368

[B46] Sampson J. A. (1927). Peritoneal endometriosis due to the menstrual dissemination of endometrial tissue into the peritoneal cavity. Am. J. Obstetr. Gynecol. 14, 422–469. 10.1016/S0002-9378(15)30003-X19969738

[B47] Thiruchelvam U. Maybin J. A. Armstrong G. M. Greaves E. Saunders P. T. Critchley H. O. (2016). Cortisol regulates the paracrine action of macrophages by inducing vasoactive gene expression in endometrial cells. J. Leukoc. Biol. 99, 1165–1171. 10.1189/jlb.5A0215-061RR 26701134 PMC4952012

[B48] Weirauch M. T. Yang A. Albu M. Cote A. G. Montenegro-Montero A. Drewe P.. (2014). Determination and inference of eukaryotic transcription factor sequence specificity. Cell 158, 1431–1443. 10.1016/j.cell.2014.08.009 25215497 PMC4163041

[B49] Yu G. Wang L. G. Han Y. He Q. Y. (2012). clusterProfiler: an R package for comparing biological themes among gene clusters. OMICS 16, 284–287. 10.1089/omi.2011.0118 22455463 PMC3339379

[B50] Zamani M. R. Salmaninejad A. Akbari Asbagh F. Masoud A. Rezaei N. (2016). STAT4 single nucleotide gene polymorphisms and susceptibility to endometriosis-related infertility. Eur. J. Obstet. Gynecol. Reprod. Biol. 203, 20–24. 10.1016/j.ejogrb.2016.05.003 27235632

